# Accelerating elimination of sleeping sickness from the Guinean littoral through enhanced screening in the post-Ebola context: A retrospective analysis

**DOI:** 10.1371/journal.pntd.0009163

**Published:** 2021-02-16

**Authors:** Oumou Camara, Sylvain Biéler, Bruno Bucheton, Moïse Kagbadouno, Joseph Mathu Ndung’u, Philippe Solano, Mamadou Camara

**Affiliations:** 1 Programme National de Lutte contre la Trypanosomiase Humaine Africaine, Conakry, Guinea; 2 Foundation for Innovative New Diagnostics (FIND), Geneva, Switzerland; 3 Institut de Recherche pour le Développement (IRD), INTERTRYP, CIRAD, Université de Montpellier, Montpellier, France; Universiteit Antwerpen, BELGIUM

## Abstract

**Background:**

Activities to control human African trypanosomiasis (HAT) in Guinea were severely hampered by the Ebola epidemic that hit this country between 2014 and 2016. Active screening was completely interrupted and passive screening could only be maintained in a few health facilities. At the end of the epidemic, medical interventions were progressively intensified to mitigate the risk of HAT resurgence and progress towards disease elimination.

**Methodology/Principal findings:**

A retrospective analysis was performed to evaluate the medical activities that were implemented in the three most endemic prefectures of Guinea (Boffa, Dubreka and Forecariah) between January 2016 and December 2018. Passive screening using rapid diagnostic tests (RDTs) was progressively resumed in one hundred and one health facilities, and active screening was intensified by visiting individual households and performing RDTs, and by conducting mass screening in villages by mobile teams using the Card Agglutination Test for Trypanosomiasis. A total of 1885, 4897 and 8023 clinical suspects were tested in passive, while 5743, 14442 and 21093 people were actively screened in 2016, 2017 and 2018, respectively. The number of HAT cases that were diagnosed first went up from 107 in 2016 to 140 in 2017, then subsequently decreased to only 73 in 2018. A progressive decrease in disease prevalence was observed in the populations that were tested in active and in passive between 2016 and 2018.

**Conclusions/Significance:**

Intensified medical interventions in the post-Ebola context first resulted in an increase in the number of HAT cases, confirming the fear that the disease could resurge as a result of impaired control activities during the Ebola epidemic. On the other hand, the decrease in disease prevalence that was observed between 2016 and 2018 is encouraging, as it suggests that the current strategy combining enhanced diagnosis, treatment and vector control is appropriate to progress towards elimination of HAT in Guinea.

## Author summary

Guinea has been consistently reporting the largest number of sleeping sickness cases in Western Africa since 2002. The Ebola epidemic that severely hit this country between 2014 and 2016 resulted in an interruption of most medical activities to control sleeping sickness, which was associated with an increase in the number of cases reported in 2016. In order to contain the resurgence of sleeping sickness and to bring it back on the path towards elimination, activities to screen and diagnose patients were intensified by strengthening the capacity of health facilities, and by visiting villages and households to test at-risk populations, while vector control was extended to cover all active foci in the country. This article presents the results that were obtained by the National Sleeping Sickness Control Program of Guinea after the Ebola epidemic by enhancing medical activities in the coastal region, where most cases of sleeping sickness are found. These efforts first resulted in an increase in the number of cases being diagnosed in 2016 and in 2017, and was then followed by an encouraging decline in 2018, thus raising hopes that the disease could be eliminated in the near future in Guinea.

## Introduction

Human African trypanosomiasis (HAT) is one of the neglected tropical diseases that are targeted for elimination as a public health problem by 2020 by the World Health Organization (WHO) [1]. Also commonly referred to as sleeping sickness, this parasitic disease is caused by two *Trypanosoma brucei* subspecies that are transmitted through the bites of tsetse flies. The large majority of cases are caused by *T*.*b*. *gambiense* infections, which are found in Western and Central Africa [1,2]. Elimination, defined as the interruption of the transmission of *T*.*b*. *gambiense* HAT has been targeted for 2030 by WHO [1].

Since 2002, Guinea has been the country reporting the largest number of HAT cases in Western Africa [1]. Most cases are found in three active foci (Boffa, Dubreka and Forécariah) that are located along the Atlantic coast, while some other cases are occasionally being reported from historical foci in the forested areas of Guinea [3,4]. The National Sleeping Sickness Control Program (NSSCP) has been coordinating efforts to control HAT in Guinea. Efforts have included both medical activities to diagnose and treat patients and thus tackle the human reservoir, and activities to control the tsetse fly vector in endemic areas [5–8].

Several new screening and diagnostic tools and methods have been developed in recent years in order to facilitate detection of HAT cases, and some of them have been introduced in Guinea, where they are being used routinely by the NSSCP. While the Card Agglutination Test for Trypanosomiasis (CATT) [9] remains the central tool for mass active screening by mobile teams, the availability of single-format rapid diagnostic tests (RDT) that detect host antibodies to trypanosomal antigens has greatly facilitated screening at an individual level, either at fixed health facilities and thus enabling better integration into primary health services [10–12], or by small teams visiting households in a so-called “door to door” strategy [13]. The HAT RDTs that are currently available for screening are the SD BIOLINE HAT and the HAT Sero-*K*-SeT, which include the same two antigens and which have been shown to have comparable diagnostic performances [14–16]. A method was also developed and introduced in Guinea to improve confirmatory diagnosis of HAT by combining the mini anion exchange centrifugation technique (mAECT) [17] with the use of buffy coat samples, resulting in higher sensitivity [18].

In early 2014, the NSSCP strengthened its capacity for passive screening by introducing HAT RDTs in 90 health posts and health centers located in the three active foci of Boffa, Dubreka and Forécariah. Unfortunately, the onset of the Ebola epidemic in 2014 [19] resulted in a complete interruption of active screening activities, as well as a disruption of most passive screening activities due to communities mistrusting the health system and to shifting priorities of the Ministry of Health towards fighting Ebola, as previously reported by Camara *et al*. [6]. In particular, screening with HAT RDTs was completely stopped in peripheral health facilities after March 2015. In addition, while confirmatory diagnosis and treatment of HAT had been available in each of the three active foci before the Ebola epidemic, these were only available in one of the foci, Dubreka, during most of the epidemic. As a result of these interruptions, the number of HAT cases that was reported annually went down from 78 in 2013 to only 33 in 2014 and 29 in 2015 [1], which probably did not reflect the true epidemiological trend of HAT during these troubled times that were characterized by very scarce and disorganized medical activities to control the disease.

With the declaration of the end of Ebola virus transmission in Guinea in June 2016, it was possible to progressively revive HAT screening activities in the most active foci in order to prevent a possible resurgence of the disease following the interruptions in medical activities, and increase the prospects for rapidly achieving its elimination in this country. We describe here a retrospective analysis of the HAT passive and active screening and diagnosis activities that were intensified by the NSSCP between 2016 and 2018, which also included the introduction of a new active screening strategy using RDTs. The objective of this work was to report on these enhanced screening activities and explore their relationship with epidemiological parameters, such as the disease prevalence, seroprevalence and disease stage at diagnosis.

## Methods

### Ethics statement

The data presented here was collected as part of the HAT elimination program in coastal Guinea (ElimTrypGui), which was approved by the Ministry of Health of the Republic of Guinea (234/MSHP/CAB/2013). All HAT patients whose data were used in this work were diagnosed and treated in agreement with the national health policy and WHO guidelines. Patient data were anonymized prior to analysis. This work was carried out in conformity with the Declaration of Helsinki.

### Setting

**Fig 1:**
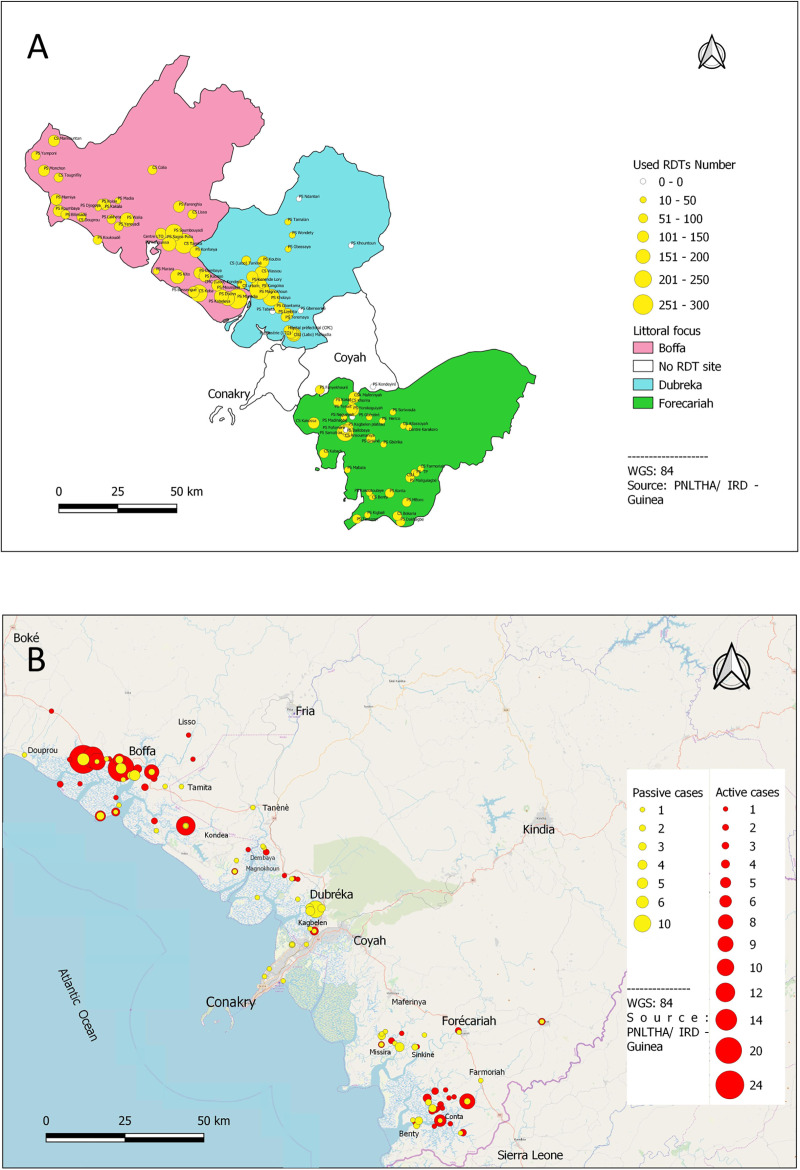
**(A) Map showing the location of health facilities performing passive screening in the Boffa, Dubreka and Forecariah prefectures.** The number of RDTs that were performed between January 2016 and December 2018 is represented by the size of the yellow discs. **(B) Map showing the location of the HAT cases that were diagnosed through active (red discs) and passive (yellow discs) screening activities between January 2016 and December 2018**.

The data that are presented here are based on passive and active screening activities that were conducted between January 2016 and December 2018 in the coastal region made of the prefectures of Boffa, Dubreka and Forecariah, which correspond to the most active HAT foci in Guinea (Fig 1). This region is characterized by a mangrove ecosystem with favorable conditions for tsetse fly vectors.

### Study design and data collection

This was a retrospective analysis of data collected by the NSSCP as part of their routine activities:

#### Passive screening

Passive screening data originated from one hundred and one health facilities (Fig 1) equipped with the SD BIOLINE HAT test (Abbott Diagnostics Korea, Republic of Korea). This RDT is an immuno-chromatographic test for qualitative detection of antibodies of all isotypes. It includes a nitrocellulose membrane strip with two test regions (T1 and T2) that are pre-coated with two trypanosome antigens and a procedural control line (C) [14,15,20]. Health workers had been previously trained to identify HAT clinical suspects to be tested with the RDT and to perform the RDT. Clinical suspects were defined as anyone presenting with at least one of the following clinical signs or symptoms: fever that persists after malaria treatment, enlarged cervical lymph nodes, behavioral change and pruritus. The RDT was performed on 20 μl of fresh blood obtained from a finger prick, according to the instructions from the manufacturer. The results from the C, T1 and T2 lines of the RDT (positive or negative) were recorded in a laboratory register. Suspects who tested positive with the RDT (defined as having a positive result with T1 or T2 or both T1 and T2) were referred to the treatment center of the corresponding prefecture for parasitological testing, staging and treatment. Meetings of personnel from all the facilities equipped with RDTs were organized by the NSSCP every two months in each prefecture, and were used to provide continuous training, discuss any operational or technical issues arising, supply more RDTs as needed and collect data. This data included the number of individuals screened with an RDT at each facility, number of RDT positive results, number of patients referred, and final diagnosis of patients.

#### Active screening

Active screening data was obtained from two different sources, depending on the screening strategy that was used. (A) Mass screening data was obtained from mobile teams conducting surveys in selected villages and screening the general population with CATT using a sample of whole blood obtained from a finger prick [9]. When the result of CATT on whole blood was positive, CATT was performed on serial dilutions of plasma as described previously [21]. Individuals with a positive CATT result on 1:4 diluted plasma were tested on site by microscopy to confirm HAT diagnosis, as described below. HAT cases were referred to the treatment center of the corresponding prefecture for staging and treatment. (B) Door to door active screening data was obtained from teams composed of one community health worker and two health workers, which visited individual households in villages that were selected based on the presence of recent HAT cases or seropositive suspects. The population was sensitized by the community health worker for two days using megaphones prior to the arrival of the screening team. In each household, inhabitants were registered and offered to be tested with an RDT, following the same procedure as described above for passive screening. When inhabitants were absent or not available at the time of the visit, they were asked to present later to the team, which stayed in the same village for several days. Confirmatory testing of RDT positive suspects was performed by a separate team composed of two health workers, who subsequently visited the relevant households to perform the microscopy methods described below. HAT cases were referred to the nearest treatment center for staging and treatment.

#### HAT confirmatory diagnosis and staging

HAT diagnosis data was collected from mobile teams in the case of patients identified by active screening, and from the following healthcare facilities in the case of passive screening: Dubreka leprosy, tuberculosis and onchocerciasis (LTO) health center (Dubreka prefecture), Karakoro health center (Forecariah prefecture) and Boffa health center (Boffa prefecture). The following procedures were performed on all serological suspects: examination of lymph node aspirate by bright field microscopy (if enlarged cervical lymph nodes were present) and mAECT on buffy coat, as previously described [18,21]. Any patient who was found to have trypanosomes using one of the above parasitological methods was staged and treated. Staging was performed according to standard procedures as described by WHO, by performing a lumbar puncture and examining the cerebrospinal fluid (CSF) by microscopy for the presence of trypanosomes and for counting white blood cells (WBCs) [21]. Patients with less than 6 WBCs per μl and with no trypanosomes in the CSF were considered as first stage cases and were treated with pentamidine, while those with more than 5 WBCs per μl and/or with trypanosomes in the CSF were considered as second stage patients and were treated with NECT, according to WHO recommendations [21]. Several patients were treated with new oral drugs (fexinidazole and acoziborole) as part of ongoing clinical trials.

### Statistical analysis

Statistical analysis was performed using Stata Statistical Software Release 12.1 [22]. Estimates expressed as percentages correspond either to proportions or to differences between two proportions, as described. Exact (Clopper-Pearson) binomial confidence intervals were computed for proportions (seroprevalences, prevalences and positive predictive values) using a significance level of 5%. Equality of proportions was tested using Stata’s *prtest* function with a confidence level of 95%.

## Results

### Passive screening

**Fig 2:**
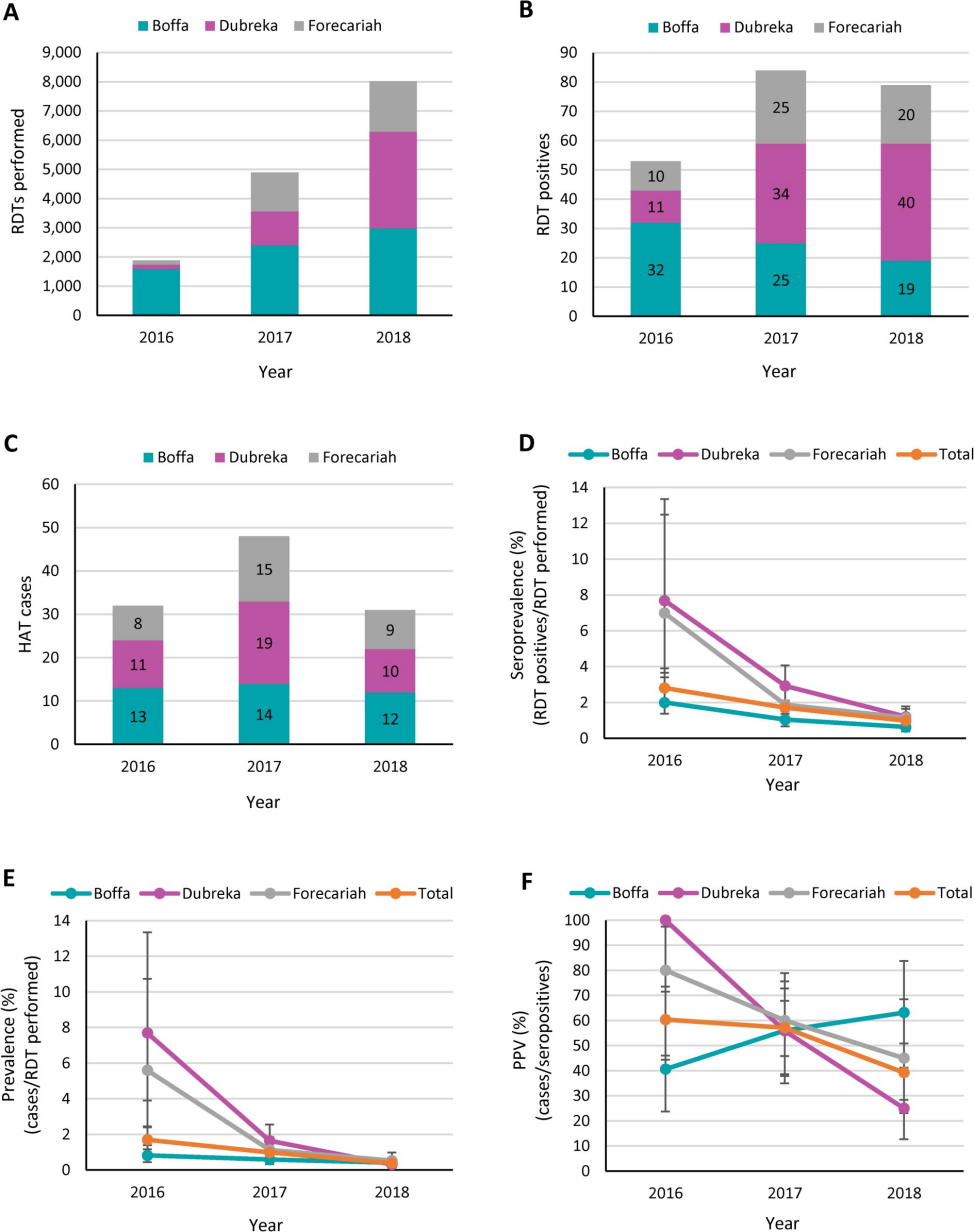
**Number of people screened with an RDT (A), number of RDT positive results (B), number of confirmed HAT cases (C), seroprevalence (D), prevalence (E) and positive predictive value (F) corresponding to the passive screening activities conducted in the Boffa, Dubreka and Forecariah prefectures between January 2016 and December 2018.** The seroprevalence was calculated as the number of RDT positive results divided by the number of people screened and expressed as a percentage. The prevalence was calculated as the number of confirmed HAT cases divided by the number of people screened and expressed as a percentage. The positive predictive value (PPV) was calculated as the number of cases divided by the number of seropositives and expressed as a percentage. Results are shown for each calendar year and for each prefecture taken individually, and as total values for the three prefectures considered together. Error bars indicate 95% confidence intervals.

Passive screening in peripheral health posts and health centers was progressively reinitiated during 2016 following the Ebola epidemic. Screening efforts were initially focused on the Boffa prefecture, as the majority of the HAT cases that were diagnosed in coastal Guinea during the Ebola outbreak originated from this prefecture. Fig 2A shows how the number of people who were tested gradually increased in each prefecture, from a total of only 1885 people in 2016 to 8023 people in 2018. While the total number of RDT positive suspects reported in the three prefectures increased significantly between 2016 and 2017 (+58.5%), it decreased slightly between 2017 and 2018 (-6.0%) (Fig 2B). Only 80.6% of the RDT positive suspects that were identified in the three prefectures during the study period were successfully referred and tested by parasitology (S1 Table). The highest referral rate was achieved in Dubreka (82.4%), while the lowest rate was reported in Forecariah (78.2%). When considering the three prefectures together, the rate of referral was almost constant between 2016 and 2017 (83.0% and 83.3%), and then went down to 75.9% in 2018. However, this decrease between 2017 and 2018 was not significant (p = 0.24). The total number of HAT cases reported from passive screening in the three prefectures first increased from 32 in 2016 to 48 in 2017, and then decreased to 31 in 2018 (Fig 2C). The distribution of cases among the prefectures varied over time, with Boffa reporting the largest number (N = 13 or 41% of all cases) in 2016, Dubreka reporting most cases in 2017 (N = 19 or 40% of all cases), and Boffa reporting again the largest number (N = 12 or 39% of all cases) in 2018. The large majority of the patients diagnosed passively were in an advanced disease stage. Only two stage 1 patients were identified in three years. Both were reported from Dubreka (in 2016 and in 2017).

The RDT seroprevalence that was reported in passive screening, expressed as the number of RDT positives identified per RDTs performed (Fig 2D), followed a similar downward pattern in each prefecture. The decrease was particularly strong in Forecariah (from 6.99% to 1.88%; p<0.001) and in Dubreka (from 7.69% to 2.93%; p = 0.003) between 2016 and 2017, while it was less pronounced between 2017 and 2018. In Boffa, there was a less strong but more steady decrease between 2016 and 2018 (from 2.00% to 0.64%; p<0.001). When considering the three prefectures together, there was a gradual decrease in seroprevalence during the study period, and there was strong evidence of a significant decrease between 2016 and 2017 (-1.10%, p = 0.004) as well as between 2017 and 2018 (-0.73%, p<0.001). The disease prevalence in passive screening, expressed as the number of HAT cases per RDTs performed (Fig 2E), followed a similar profile as the seroprevalence. There was a strong decrease in prevalence in Forecariah (-4.47%, p<0.001) and in Dubreka (-6.05%, p<0.001) between 2016 and 2017, while the decrease was less marked between 2017 and 2018, and in the Boffa prefecture. When considering the 3 prefectures together, there was strong evidence of a significant decrease in HAT prevalence between 2016 and 2017 (-0.72%, p = 0.014) as well as between 2017 and 2018 (-0.59%, p<0.001). The average positive predictive value (PPV) of the RDT in passive screening (Fig 2F) was 51.4% when considering the three prefectures together throughout the study period, and it progressively decreased between 2016 and 2018 (from 60.4% to 39.2%, p = 0.017). There appeared to be an exception in Boffa, where the PPV gradually increased during this period, but this increase was not significant (from 40.6% to 63.2%, p = 0.12).

### Active screening

**Fig 3:**
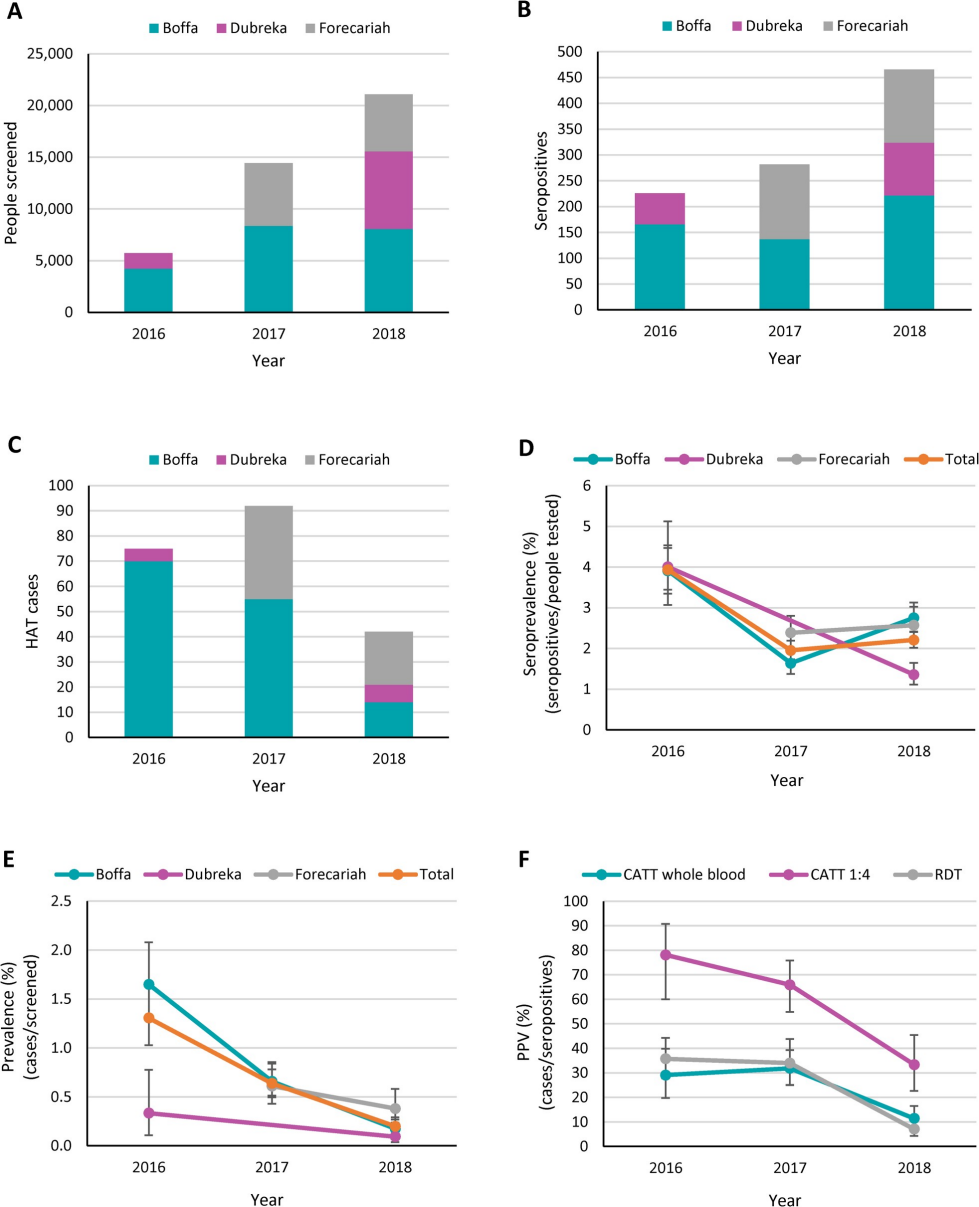
**Number of people screened either with an RDT or with CATT (A), number of seropositives (B), number of confirmed HAT cases (C), seroprevalence (D), prevalence (E) and positive predictive value (F) corresponding to the active screening activities conducted in the Boffa, Dubreka and Forecariah prefectures between January 2016 and December 2018.** Seropositives include all the individuals found positive with an RDT or with CATT performed on whole blood. The seroprevalence was calculated as the number of seropositives divided by the number of people screened and expressed as a percentage. The prevalence was calculated as the number of confirmed HAT cases divided by the number of people screened and expressed as a percentage. The positive predictive value (PPV) was calculated as the number of cases divided by the number of seropositives and expressed as a percentage. Results from each calendar year are shown for each prefecture taken individually and as total values for the three prefectures considered together (A-E), or for each type of serological test (F). CATT 1:4 refers to the results that were obtained when CATT was performed on 1:4 diluted plasma. Error bars indicate 95% confidence intervals.

Active screening during the study period included both the classical mass screening strategy with CATT and the door to door screening strategy using RDTs. In 2016, active screening consisted of mass screening campaigns that were organized in the Boffa prefecture, as well as door to door screening in the Boffa and Dubreka prefectures. In 2017, mass screening in the Boffa and Forecariah prefectures was complemented by door to door screening in the same prefectures. In 2018, both mass screening and door to door screening were conducted in each of the three prefectures (S2 Table). While the total number of people who were actively screened in these prefectures was only 5,743 in 2016 as the country was still recovering from the Ebola crisis, it sharply increased to 14,442 in 2017 and to 21,093 people in 2018 (Fig 3A). The number of seropositives (considering both positive results obtained with CATT on whole blood or with RDTs) also went up between 2016 and 2018, but to a lesser extent than the number of people tested (2.06-fold increase in the number of seropositives, as compared to a 3.67-fold increase in the number of people tested) (Fig 3B). All the seropositives that were identified by active screening were tested by microscopy. Like with the HAT cases detected through passive screening, the number of cases diagnosed through active screening first went up from 75 in 2016 to 92 in 2017, and then decreased to only 42 in 2018 (Fig 3C). The proportion of HAT cases diagnosed in the second stage of the disease in the three prefectures was relatively low in 2016 and in 2017 (70.7% in both years) and went up in 2018 (88.1%). The increase between 2017 and 2018 was significant (p = 0.028). Five (2.4%) out of the 209 HAT cases that were diagnosed through active screening between 2016 and 2018 could not be staged, as they refused to undergo a lumbar puncture (S2 Table).

The seroprevalence that was reported in active screening, calculated as the number of seropositives (CATT on whole blood or RDTs) obtained out of the total people screened (Fig 3D), followed a different pattern in each prefecture. In Boffa, the seroprevalence first decreased between 2016 and 2017 (-2.27%, p<0.001) and then went up again between 2017 and 2018 (+1.11%, p<0.001). In Dubreka, active screening was only conducted in 2016 and in 2018 and comparing the respective seroprevalence values indicated a significant decrease (-2.65%; p<0.001). Seroprevalence data from Forecariah was only available in 2017 and 2018, when it remained relatively stable (+0.18%; p = 0.52). When considering the three prefectures together, there was strong evidence for a significant decrease between 2016 and 2017 (-1.98%, p<0.001), while the seroprevalence remained quite stable between 2017 and 2018 (+0.26%; p = 0.098).

Fig 3E shows that the disease prevalence progressively decreased in each prefecture between 2016 and 2018. In particular, there was a strong decrease in Boffa between 2016 and 2017 (-0.99%; p<0.001) as well as between 2017 and 2018 (-0.48%; p<0.001). There was also a significant but less pronounced decrease in the prevalence in Dubreka between 2016 and 2018 (-0.24%; p = 0.020) but no evidence of any significant decrease in Forecariah between 2017 and 2018 (-0.23%; p = 0.081). When considering the 3 prefectures together, there was strong evidence of a significant decrease in HAT prevalence between 2016 and 2017 (-0.67%, p<0.001) as well as between 2017 and 2018 (-0.44%, p<0.001).

The PPV of each test that was used in active screening decreased during the study period (Fig 3F). The highest PPV was achieved by performing the CATT test on 1:4 diluted plasma, which went down from 78.1% in 2016 to 33.3% in 2018 (p<0.001). The PPV of the CATT test performed on whole blood and of the RDT were lower and decreased from 29.1% to 11.4% (p<0.001) and from 35.7% to 7.1% (p<0.001), respectively, between 2016 and 2018.

Both mass screening and door to door screening activities were conducted in the three prefectures in 2018, allowing for a comparison of the results obtained with the two screening strategies (Table 1). The seroprevalence that was obtained by door to door screening with the RDT (2.69%) was significantly higher than by mass screening with CATT (1.82%) (p<0.001). However, there was no evidence of a significant difference in the HAT prevalence (p = 0.78) or in the PPV (p = 0.11) between these two screening strategies.

**Table 1:** Comparison of the results obtained in mass screening and in door to door screening in 2018 in the Boffa, Dubreka and Forecariah prefectures.

	Mass screening (CATT whole blood)	Door to door screening (RDT)	P value
**Population screened**	11,603	9,490	
**Seropositives**	211	255	
**Seroprevalence (%)**	1.82	2.69	<0.001
**HAT cases**	24	18	
**Prevalence (%)**	0.21	0.19	0.78
**PPV (%)**	11.4	7.1	0.11

### Progress towards elimination

Elimination of HAT as a public health problem has been defined by WHO as being achieved when less than one new case is reported per 10,000 inhabitants and per year in 90% of foci [21]. Table 2 shows that while in 2018 there were still 1.1 cases per 10,000 inhabitants reported in Boffa, there was less than 1 case per 10,000 reported in the other prefectures (0.47 in Dubreka and 0.87 in Forecariah), as well as in the three prefectures considered together (0.78). This constitutes an improvement in comparison to the numbers of cases per 10,000 inhabitants that were reported in 2017, which were higher in the three prefectures (2.92 in Boffa, 0.53 in Dubreka and 1.51 in Forecariah). In 2016, this number was even higher in Boffa (3.52 cases per 10,000 inhabitants), but it was very low in Dubreka (0.44) and in Forecariah (0.23) since there were limited screening activities in these prefectures. In 2019, the number of cases per 10,000 inhabitants was finally lower than 1 in each of the three prefectures, and was lower than in 2018 when considering the three prefectures together (0.73).

**Table 2:** HAT cases reported in the Boffa, Dubreka and Forecariah prefectures in 2016, 2017, 2018 and 2019, expressed as absolute numbers and as numbers per 10,000 inhabitants.

		2016		2017		2018		2019	
	Population	Cases	Cases / 10,000 inhabitants	Cases	Cases / 10,000 inhabitants	Cases	Cases / 10,000 inhabitants	Cases	Cases / 10,000 inhabitants
Boffa	235,903	83	3.52	69	2.92	26	1.10	23	0.97
Dubreka	359,888	16	0.44	19	0.53	17	0.47	12	0.33
Forecariah	343,292	8	0.23	52	1.51	30	0.87	34	0.99
Total	939,083	107	1.14	140	1.49	73	0.78	69	0.73

The total population of each prefecture was obtained from the NSSCP (personal communication). The numbers of cases include cases identified through both active and passive screening activities. The cases reported in the Dubreka prefecture include a few patients who were living in Conakry at the time of diagnosis (2, 3 and 1 in 2017, 2018 and 2019, respectively), but who are mostly believed to have been infected in the Dubreka prefecture.

## Discussion

This study has shown that there was a progressive intensification of HAT screening and diagnosis activities between 2016 and 2018 in coastal Guinea, following the Ebola outbreak that hit this region between 2014 and early 2016. This in itself was already a remarkable achievement by the Ministry of Health of Guinea, the NSSCP and their collaborators and partners in the field, who were successful in re-establishing a large network of health facilities equipped with RDTs and organising increasingly frequent active screening campaigns in this HAT endemic region. It was also a strong positive signal showing that the communities that had been so severely affected by the Ebola crisis were progressively regaining trust in the health system and were again willing to be tested for HAT, which had been extremely challenging just a few years earlier [6].

The observation that the progressive increase in the number of people tested in passive screening that occurred in the three prefectures was only mirrored by an increase in the number of seropositives between 2016 and 2017, but that the number of seropositives decreased between 2017 and 2018, could already suggest a trend towards a decline of the disease in this region, as evidenced by the progressive decrease in the overall seroprevalence that was observed in passive screening during the study period. However, seroprevalence data should be interpreted with caution, as seropositives could correspond to at least four different categories of positive results, which would be difficult to untangle here. Some seropositives obviously correspond to true positives that were confirmed as HAT cases by microscopy. Other seropositives that were not confirmed by microscopy could also be true positives that were not successfully identified and treated as such, since the microscopy procedures that were performed are known to occasionally miss some cases, even when several procedures are combined [18,21,23]. In addition, a significant number of seropositives were not successfully referred and tested by microscopy, and therefore, their true nature is unknown. Finally, some seropositives are likely to be false positives due to the imperfect specificity of the SD BIOLINE HAT RDT [14,15,20], which could also possibly cross-react with other parasites [24]. Although the relative importance of these different categories of seropositives cannot be reliably determined in the present study, and interpreting seroprevalence data is therefore challenging, the observation that the overall seroprevalence declined during this study indicates that the percentage of the population harbouring antibodies against the trypanosomal antigens used in the RDT declined, which would at least be consistent with the view that progress was made towards disease elimination. However, one could not exclude other explanations for this decline in seroprevalence, such as a decrease in the prevalence of other parasites that may cross-react with the RDT.

A more direct evidence of progress towards disease elimination during the study period was provided by the number of HAT cases that were identified through passive screening activities and the corresponding disease prevalence trend. While the intensification of screening activities first resulted in an overall increase in the number of cases between 2016 and 2017, there was a concomitant decline in the prevalence of HAT between these two years, which was maintained between 2017 and 2018. Strikingly, the same observations were made in each of the three prefectures, thus strengthening the view that real progress was made towards disease elimination in the Guinean littoral.

The fact that the large majority of cases diagnosed through passive screening were identified when they were already in the second stage of the disease could be interpreted in two different ways, considering that *T*.*b*. *gambiense* HAT is a chronic disease that can last for months or even years [25,26]. One could be tempted to conclude that since cases had been infected for rather long, there was little active transmission in the region. On the other hand, it would also be possible that first-stage cases existed but did not present at health facilities for screening, since their clinical status was not advanced enough to prevent them from undertaking usual daily activities. Such individuals were probably not very concerned by their health status and therefore not keen to travel a long distance for further medical investigations, in particular considering the recent Ebola crisis. This second interpretation would be in agreement with the observation that a significant number of seropositives were not successfully referred for confirmatory testing and would be supported by the finding that a lower percentage of second-stage cases was reported through active screening activities in the same region. Thus, although transmission seems to have significantly decreased during the study period, the fact that a few first-stage cases were still diagnosed in 2018 by active screening may challenge the view that the medical and vector control activities that had been deployed in this region would be sufficient to achieve disease elimination. Since some of these activities, in particular vector control, were only recently introduced in some of the prefectures, monitoring the number and stage of the cases that will be identified in the coming years will be key to determine if disease control activities are adequate, or if active transmission is still occurring in this region.

The overall PPV that was found with the SD BIOLINE HAT RDT in passive screening during the study period (51.4%) was higher than expected. In a previous study that was conducted in the Democratic Republic of the Congo (DRC), the PPV of this RDT was estimated to be only 24.4% with a disease prevalence of 1% [20], while the average prevalence that was observed in passive screening in the present study was only 0.75%. Several hypotheses could be formulated to explain this apparent discrepancy. One possibility would be that in Guinea, healthcare workers may tend to only interpret RDTs with strongly visible test lines as positive, while in the earlier study, all visible test lines, including faint ones, were interpreted as positive. Consequently, and assuming that blood samples from true HAT cases would exhibit a stronger reaction to the RDT antigens than false positives, which is possible but would need to be formally demonstrated, the likelihood of reporting false positive results would be lower in Guinea. Although there is no direct reason to believe that there was such a difference between Guinean healthcare workers and their Congolese counterparts regarding the interpretation of RDT results, it is likely that the level of training and supervision was higher in the earlier study, which was a prospective clinical study that was conducted to evaluate the diagnostic accuracy of the RDT, as opposed to the routine disease control activities that were organised in Guinea. Another potential explanation, which is however not supported by any available data, could be that the RDT would be more specific in the Guinean population due to differences in host factors or in epidemiological parameters such as the prevalence of other pathogens that would cause cross-reactions with the RDT antigens. This difference in PPV between DRC and Guinea could also be due to differences in the sensitivity of confirmation tests. In Guinea for example the majority of patients (>70%) are positive to lymph node aspirate (LNA) examination [18] whereas this proportion was shown to be lower in DRC in the Masi-Manimba focus [23]. Another difference is that in Guinea the microscopic examination of blood with mAECT is performed using buffy coat samples instead of whole blood, which increases the sensitivity of trypanosome detection [18,21]. It is noteworthy however that the observed PPV of the SD BIOLINE HAT RDT in active screening was much lower (20.8% over the study period) and more similar to what was reported in the DRC study. This would thus indicate that in the peripheral health facilities of Guinea the SD BIOLINE HAT RDT was mainly performed on subjects with severe symptoms, as also suggested by the very high proportion of stage 2 patients diagnosed. It is also likely that the proportion of false positive serology was higher in the subjects who, not feeling ill, did not present for parasitological confirmation, thus introducing a bias resulting in an higher PPV of the SD BIOLINE HAT RDT in passive screening.

The observation that the PPV of passive screening gradually decreased during the study period could be explained by the concomitant decline in disease prevalence. The fact that in 2018 the majority of RDT positive suspects were false positives highlights some limitations of the current passive screening approach, which are likely to become more critical in the coming years if the prevalence continues to go down. In particular, one could expect that the health personnel will become more doubtful about the validity of RDT results, and that it will be increasingly difficult to convince RDT positive suspects to travel for confirmatory testing. Therefore, in order to ensure an adequate level of testing and successful referral, continuous training and supervision of healthcare workers as well as advocacy efforts targeting communities will become increasingly important. Alternatively, in order to increase the PPV of screening, one option might be to combine the use of several different RDTs in diagnostic algorithms (including those that are currently available and new RDTs that are being developed), provided that this does not result in a significant drop in sensitivity.

The fact that about one fifth of the RDT positive suspects that were identified in health facilities were not tested by microscopy is a limitation of the passive case detection system that has been established in Guinea. Similar challenges were faced in Uganda, where a referral rate of 79.9% was reported after introducing HAT RDTs in primary healthcare facilities [10]. Although some of the cases that may have been missed could be diagnosed later when they have a more advanced form of the disease, these undetected cases would in the meantime represent a significant threat to disease elimination efforts, and complementary strategies may therefore need to be implemented to ensure that they are kept to a minimum. The NSSCP has been exploring options to improve referral, such as taking advantage of targeted active screening activities like door to door screening to screen these seropositives again and try to convince them to undergo confirmatory testing, as well as raising awareness of healthcare workers and communities on HAT and the importance of early diagnosis. Another option might be to use a small mobile team to look for these seropositives and test them by microscopy. Alternatively, a strategy that could be used in the future, if new drugs that are sufficiently safe and easy to administer are made available, would be to treat all the seropositives without referring them for confirmatory testing [27].

The results that were obtained through active screening activities essentially led to the same conclusions as those from passive screening. Active screening activities were progressively intensified, which resulted in an increasing number of seropositives being identified. There was also a downward trend in the seroprevalence and in the disease prevalence during the study period. In addition, the observation that the proportion of cases with recent infections decreased during the study period, as evidenced by the observed increase in the percentage of cases that were diagnosed in the second stage of the disease in active screening between 2017 and 2018, would support the view that disease control interventions (including medical and vector control activities) had a significant impact on disease transmission. However, since different villages within each prefectures were screened in different years, potential differences between the corresponding populations could also explain these results, including various degrees of exposure to tsetse flies, which could not be assessed in the present study. Such differences between populations could also provide an explanation for the unexpected increase in seroprevalence that was reported in Boffa between 2017 and 2018, which was not observed through passive screening.

Similarly to what was observed with passive screening, the low PPV that was reported in 2018 with each of the serological tests that were used in active screening would be a direct consequence of the declining prevalence in this population. Even when considering 1:4 diluted plasma as cut-off, two thirds of the serological suspects that were identified with CATT in 2018 were negative by microscopy. With an increasingly large percentage of seropositives being false positives but still requiring to go through parasitological confirmation, the suitability of active screening could be challenged. Indeed, the high workload for healthcare workers to perform confirmatory testing and the associated discomfort and waste of time for patients are likely to become increasingly difficult to justify and therefore result in growing resistance to these active screening strategies. In order to adapt to these changes and improve population coverage in high risk areas, the Guinean NSSCP is progressively switching from the classical mass screening strategies using CATT to new active screening strategies that are integrated in the peripheral health system, in which the local healthcare workers involved in passive surveillance perform active door to door screening with RDTs in the most affected villages. Mass screening campaigns are still maintained in the most active foci (Forecariah and Boffa), as they enable visiting a large number of villages and contribute to mapping efforts to identify the most affected villages where screening should be intensified. It is also worth noting that the proportion of patients diagnosed through the passive surveillance system increased from 29.9% in 2016 to 42.5% in 2018, indicating that the performance of this system improved during the study period. Thus, although passive surveillance usually detects patients in an advanced disease stage, passive screening appears very complementary to active screening in Guinea. As shown in Fig 1B there was also a good overlap between the distributions of cases identified through active and passive screening, in particular in Boffa and in Forecariah. This suggests that infected individuals who would have been missed by active screening teams eventually presented to nearby health facilities when their health status deteriorated, thus contributing to the reduction of the human reservoir of trypanosomes.

The fact that door to door active screening resulted in a higher seroprevalence than classical mass screening is in agreement with the view that door to door screening is more targeted to high-risk populations, although we could not observe in the present study any significant difference in the disease prevalence between the populations that were tested with these different screening strategies. However, directly comparing these two populations could be questionable, as a number of villages that were screened using the door to door strategy were selected because HAT cases had been previously detected during mass screening. Further investigations will therefore be necessary to confirm whether door to door screening is more effective in finding HAT cases than mass screening in the Guinean context, as previously found in Côte d’Ivoire [13]. Comparing results from Côte d’Ivoire and from Guinea could also be difficult, as the door to door screening strategy that was used in Côte d’Ivoire was somehow different, with screening being only targeted to the close neighborhood of previous HAT patients or serological suspects. This is in contrast to what was done in Guinea, where door to door screening was organized to cover entire villages in order to achieve a maximum population coverage in high risk areas.

The finding that the results obtained in active and in passive screening, which were based on different population samples, exhibited similar epidemiological trends would further support the view that real progress was made towards disease elimination between 2016 and 2018. Although a causal relationship between the increase in active and passive screening activities and the observed prevalence decline cannot be formally demonstrated with the present study, it seems very likely, as diagnosing and treating cases and thus reducing the human reservoir is generally accepted as the central strategy to control *T*.*b*. *gambiens*e HAT [2,21]. However, the contribution of vector control interventions in the study area should not be overlooked. In particular, the deployment of small insecticide impregnated targets (“tiny targets”) in active foci appears to have played a key role in reducing *T*.*b*. *gambiense* transmission. Deployment of tiny targets was initiated in 2012 in the eastern part of the Boffa prefecture, where it was shown to speed up the elimination process [5,8]. Since then, tiny targets have been replaced every year in this area, even during the Ebola outbreak. Strikingly, the HAT prevalence that was reported by active screening in the eastern part of the Boffa prefecture in 2016 was much lower than in the western part of the prefecture, where the disease prevalence reached epidemic levels in the most affected villages [28]. Tiny target deployment was subsequently extended to the whole Boffa focus and to the Dubreka focus in 2016, as well as to the Forecariah focus in 2018. In 2018, a total of 16,079 tiny targets were deployed in the three foci and a mean 72% reduction in tsetse densities was observed at the 91 sentinel sites used for entomological evaluation, in comparison to the baseline densities that were measured prior to the initial deployments (S1 Text). Interestingly, the lowest HAT prevalence values that were found in 2018 by active screening were in Boffa and in Dubreka (0.17% and 0.09%, respectively), where vector control activities had been implemented for the longest period (at least 2 years). By contrast, a higher prevalence was reported in the same year by active screening in Forecariah (0.38%), where tiny targets had been deployed for a shorter duration (less than 1 year).

During the two years that preceded the Ebola outbreak (2012–2013), 104 HAT cases were diagnosed through mass screening of 32,221 people in coastal Guinea [6], corresponding to a prevalence of 0.32%. The significantly higher prevalence that was observed in active screening in the year that followed the Ebola outbreak (2016) in our study (1.31%, p<0.001) is consistent with an increase in disease transmission during the Ebola period (2014–2015) as a result of interruptions of medical activities, as hypothesized by Camara *et al*. [6]. Our data have shown that this trend was successfully reverted in 2017 and in 2018 (with a prevalence by active screening of 0.64% and 0.2%, respectively), when both medical and vector control activities were intensified. This trend, together with the observations that the number of new cases reported has been close to one per 10,000 inhabitants in each prefecture during the study period and that the total number of cases reported nationally has been gradually decreasing from 2017 to 2019, are strong signs that elimination of HAT as a public health problem in Guinea could be achieved in the near future.

## Supporting information

S1 Table Results of passive screening activities conducted in the Boffa, Dubreka and Forecariah prefectures between January 2016 and December 2018. The seroprevalence was calculated as the number of RDT positive results divided by the number of people screened and expressed as a percentage. The referral rate was calculated as the number of seropositives that were tested by microscopy divided by the total number of seropositives and expressed as a percentage. The prevalence was calculated as the number of confirmed HAT cases divided by the number of people screened and expressed as a percentage. The positive predictive value (PPV) was calculated as the number of cases divided by the number of RDT positives and expressed as a percentage. Results are shown for each calendar year and for each prefecture.
(DOCX)

S2 Table Results of active screening activities conducted in the Boffa, Dubreka and Forecariah prefectures between January 2016 and December 2018. The population screened (total) refers to people tested either by mass screening with CATT or by door to door screening with RDTs. Seropositives (total) include all the individuals found positive with CATT performed on whole blood or with an RDT. Seropositives (CATT 1:4) include all the individuals found positive with CATT performed on 1:4 diluted plasma. The seroprevalence was calculated as the total number of seropositives divided by the total number of people screened and expressed as a percentage. The prevalence was calculated as the number of confirmed HAT cases divided by the total number of people screened and expressed as a percentage. The positive predictive value (PPV) was calculated as the number of cases divided by the number of seropositives identified either with CATT performed on whole blood, CATT performed on 1:4 diluted plasma or with an RDT, and expressed as a percentage. Results are shown for each calendar year and for each prefecture.
(DOCX)

S1 Text Annual tiny target deployments in the HAT active foci of Guinea. (DOCX)
