# Supplementary material for: Accelerating elimination of sleeping sickness from the Guinean littoral through enhanced screening in the post-Ebola context: A retrospective analysis
Source: PLoS Negl Trop Dis. 2021 Feb 16;15(2):e0009163. doi: 10.1371/journal.pntd.0009163 (PMC7909630; doi:10.1371/journal.pntd.0009163)
Supplement: S1 Table — The seroprevalence was calculated as the number of RDT positive results divided by the number of people screened and expressed as a percentage. The referral rate was calculated as the number of seropositives that were tested by microscopy divided by the total number of seropositives and expressed as a percentage. The prevalence was calculated as the number of confirmed HAT cases divided by the number of people screened and expressed as a percentage. The positive predictive value (PPV) was calculated as the number of cases divided by the number of RDT positives and expressed as a percentage. Results are shown for each calendar year and for each prefecture. (DOCX) [file pntd.0009163.s001.docx]

**S1 Table. Results of passive screening activities conducted in the Boffa, Dubreka and Forecariah prefectures between January 2016 and December 2018.**

|  | **Prefecture** | **2016** | **2017** | **2018** | **Total** |
| --- | --- | --- | --- | --- | --- |
| **Population screened with an RDT** | Boffa | 1,599 | 2,404 | 2,985 | 6,988 |
|  | Dubreka | 143 | 1,160 | 3,307 | 4,610 |
|  | Forecariah | 143 | 1,333 | 1,731 | 3,207 |
|  | Total | 1,885 | 4,897 | 8,023 | 14,805 |
| **Seropositives** | Boffa | 32 | 25 | 19 | 76 |
|  | Dubreka | 11 | 34 | 40 | 85 |
|  | Forecariah | 10 | 25 | 20 | 55 |
|  | Total | 53 | 84 | 79 | 216 |
| **Seroprevalence (%)** | Boffa | 2.00 | 1.04 | 0.64 | 1.09 |
|  | Dubreka | 7.69 | 2.93 | 1.21 | 1.84 |
|  | Forecariah | 6.99 | 1.88 | 1.16 | 1.71 |
|  | Total | 2.81 | 1.72 | 0.98 | 1.46 |
| **Seropositives tested by microscopy (n)** | Boffa | 23 | 21 | 17 | 61 |
|  | Dubreka | 11 | 29 | 30 | 70 |
|  | Forecariah | 10 | 20 | 13 | 43 |
|  | Total | 44 | 70 | 60 | 174 |
| **Referral rate (%)** | Boffa | 71.9 | 84.0 | 89.5 | 80.3 |
|  | Dubreka | 100.0 | 85.3 | 75.0 | 82.4 |
|  | Forecariah | 100.0 | 80.0 | 65.0 | 78.2 |
|  | Total | 83.0 | 83.3 | 75.9 | 80.6 |
| **HAT cases** | Boffa | 13 | 14 | 12 | 39 |
|  | Dubreka | 11 | 19 | 10 | 40 |
|  | Forecariah | 8 | 15 | 9 | 32 |
|  | Total | 32 | 48 | 31 | 111 |
| **Prevalence (%)** | Boffa | 0.81 | 0.58 | 0.40 | 0.56 |
|  | Dubreka | 7.69 | 1.64 | 0.30 | 0.87 |
|  | Forecariah | 5.59 | 1.13 | 0.52 | 1.00 |
|  | Total | 1.70 | 0.98 | 0.39 | 0.75 |
| **Stage 1 cases (n)** | Boffa | 0 | 0 | 0 | 0 |
|  | Dubreka | 1 | 1 | 0 | 2 |
|  | Forecariah | 0 | 0 | 0 | 0 |
|  | Total | 1 | 1 | 0 | 2 |
| **Stage 2 cases (n)** | Boffa | 12 | 14 | 12 | 38 |
|  | Dubreka | 9 | 18 | 9 | 36 |
|  | Forecariah | 8 | 14 | 9 | 31 |
|  | Total | 29 | 46 | 30 | 105 |
| **Cases with unknown stage (n)** | Boffa | 1 | 0 | 0 | 1 |
|  | Dubreka | 1 | 0 | 1 | 2 |
|  | Forecariah | 0 | 1 | 0 | 1 |
|  | Total | 2 | 1 | 1 | 4 |
| **Stage 2 cases (%)** | Boffa | 92.3 | 100.0 | 100.0 | 97.4 |
|  | Dubreka | 81.8 | 94.7 | 90.0 | 90.0 |
|  | Forecariah | 100.0 | 93.3 | 100.0 | 96.9 |
|  | Total | 90.6 | 95.8 | 96.8 | 94.6 |
| **PPV (%)** | Boffa | 40.6 | 56.0 | 63.2 | 51.3 |
|  | Dubreka | 100.0 | 55.9 | 25.0 | 47.1 |
|  | Forecariah | 80.0 | 60.0 | 45.0 | 58.2 |
|  | Total | 60.4 | 57.1 | 39.2 | 51.4 |
