# Supplementary material for: Accelerating elimination of sleeping sickness from the Guinean littoral through enhanced screening in the post-Ebola context: A retrospective analysis
Source: PLoS Negl Trop Dis. 2021 Feb 16;15(2):e0009163. doi: 10.1371/journal.pntd.0009163 (PMC7909630; doi:10.1371/journal.pntd.0009163)
Supplement: S2 Table — The population screened (total) refers to people tested either by mass screening with CATT or by door to door screening with RDTs. Seropositives (total) include all the individuals found positive with CATT performed on whole blood or with an RDT. Seropositives (CATT 1:4) include all the individuals found positive with CATT performed on 1:4 diluted plasma. The seroprevalence was calculated as the total number of seropositives divided by the total number of people screened and expressed as a percentage. The prevalence was calculated as the number of confirmed HAT cases divided by the total number of people screened and expressed as a percentage. The positive predictive value (PPV) was calculated as the number of cases divided by the number of seropositives identified either with CATT performed on whole blood, CATT performed on 1:4 diluted plasma or with an RDT, and expressed as a percentage. Results are shown for each calendar year and for each prefecture. (DOCX) [file pntd.0009163.s002.docx]

**S2 Table. Results of active screening activities conducted in the Boffa, Dubreka and Forecariah prefectures between January 2016 and December 2018.**

|  | **Prefecture** | **2016** | **2017** | **2018** | **Total** |
| --- | --- | --- | --- | --- | --- |
| **Population screened (total)** | Boffa | 4,245 | 8,369 | 8,071 | 20,685 |
|  | Dubreka | 1,498 | - | 7,502 | 9,000 |
|  | Forecariah | - | 6,073 | 5,520 | 11,593 |
|  | Total | 5,743 | 14,442 | 21,093 | 41,278 |
| **Population screened (CATT)** | Boffa | 2,859 | 7,230 | 5,179 | 15,268 |
|  | Dubreka | - | - | 3,263 | 3,263 |
|  | Forecariah | - | 4,661 | 3,161 | 7,822 |
|  | Total | 2,859 | 11,891 | 11,603 | 26,353 |
| **Population screened (RDT)** | Boffa | 1,386 | 1,139 | 2,892 | 5,417 |
|  | Dubreka | 1,498 | - | 4,239 | 5,737 |
|  | Forecariah | - | 1,412 | 2,359 | 3,771 |
|  | Total | 2,884 | 2,551 | 9,490 | 14,925 |
| **Seropositives (total)** | Boffa | 166 | 137 | 222 | 525 |
|  | Dubreka | 60 | - | 102 | 162 |
|  | Forecariah | - | 145 | 142 | 287 |
|  | Total | 226 | 282 | 466 | 974 |
| **Seropositives (CATT whole blood)** | Boffa | 86 | 100 | 116 | 302 |
|  | Dubreka | - | - | 46 | 46 |
|  | Forecariah | - | 76 | 49 | 125 |
|  | Total | 86 | 176 | 211 | 473 |
| **Seropositives (CATT 1:4)** | Boffa | 32 | 68 | 30 | 130 |
|  | Dubreka | - | - | 18 | 18 |
|  | Forecariah | - | 17 | 24 | 41 |
|  | Total | 32 | 85 | 72 | 189 |
| **Seropositives (RDT)** | Boffa | 80 | 37 | 106 | 223 |
|  | Dubreka | 60 | - | 56 | 116 |
|  | Forecariah | - | 69 | 93 | 162 |
|  | Total | 140 | 106 | 255 | 501 |
| **Seroprevalence (%)** | Boffa | 3.91 | 1.64 | 2.75 | 2.54 |
|  | Dubreka | 4.01 | - | 1.36 | 1.80 |
|  | Forecariah | - | 2.39 | 2.57 | 2.48 |
|  | Total | 3.94 | 1.95 | 2.21 | 2.36 |
| **HAT cases (total)** | Boffa | 70 | 55 | 14 | 139 |
|  | Dubreka | 5 | - | 7 | 12 |
|  | Forecariah | 0 | 37 | 21 | 58 |
|  | Total | 75 | 92 | 42 | 209 |
| **HAT cases (CATT)** | Boffa | 25 | 44 | 10 | 79 |
|  | Dubreka | - | - | 3 | 3 |
|  | Forecariah | - | 12 | 11 | 23 |
|  | Total | 25 | 56 | 24 | 105 |
| **HAT cases (RDT)** | Boffa | 45 | 11 | 4 | 60 |
|  | Dubreka | 5 | - | 4 | 9 |
|  | Forecariah | - | 25 | 10 | 35 |
|  | Total | 50 | 36 | 18 | 104 |
| **Prevalence (%)** | Boffa | 1.65 | 0.66 | 0.17 | 0.67 |
|  | Dubreka | 0.33 | - | 0.09 | 0.13 |
|  | Forecariah | - | 0.61 | 0.38 | 0.50 |
|  | Total | 1.31 | 0.64 | 0.20 | 0.51 |
| **Stage 1 cases (n)** | Boffa | 19 | 15 | 0 | 34 |
|  | Dubreka | 1 | - | 1 | 2 |
|  | Forecariah | - | 11 | 2 | 13 |
|  | Total | 20 | 26 | 3 | 49 |
| **Stage 2 cases (n)** | Boffa | 50 | 40 | 14 | 104 |
|  | Dubreka | 3 | - | 5 | 8 |
|  | Forecariah | - | 25 | 18 | 43 |
|  | Total | 53 | 65 | 37 | 155 |
| **Cases with unknown stage (n)** | Boffa | 1 | 0 | 0 | 1 |
|  | Dubreka | 1 | 0 | 1 | 2 |
|  | Forecariah | 0 | 1 | 1 | 2 |
|  | Total | 2 | 1 | 2 | 5 |
| **Stage 2 cases (%)** | Boffa | 71.4 | 72.7 | 100.0 | 74.8 |
|  | Dubreka | 60.0 | - | 71.4 | 66.7 |
|  | Forecariah | - | 67.6 | 85.7 | 74.1 |
|  | Total | 70.7 | 70.7 | 88.1 | 74.2 |
| **PPV CATT whole blood (%)** | Boffa | 29.1 | 44.0 | 8.6 | 26.2 |
|  | Dubreka | - | - | 6.5 | 6.5 |
|  | Forecariah | - | 15.8 | 22.4 | 18.4 |
|  | Total | 29.1 | 31.8 | 11.4 | 22.2 |
| **PPV CATT 1:4 (%)** | Boffa | 78.1 | 64.7 | 33.3 | 60.8 |
|  | Dubreka | - | - | 16.7 | 16.7 |
|  | Forecariah | - | 70.6 | 45.8 | 56.1 |
|  | Total | 78.1 | 65.9 | 33.3 | 55.6 |
| **PPV RDT (%)** | Boffa | 56.3 | 29.7 | 3.8 | 26.9 |
|  | Dubreka | 8.3 | - | 7.1 | 7.8 |
|  | Forecariah | - | 36.2 | 10.8 | 21.6 |
|  | Total | 35.7 | 34.0 | 7.1 | 20.8 |
