# Supplementary material for: Accelerating elimination of sleeping sickness from the Guinean littoral through enhanced screening in the post-Ebola context: A retrospective analysis
Source: PLoS Negl Trop Dis. 2021 Feb 16;15(2):e0009163. doi: 10.1371/journal.pntd.0009163 (PMC7909630; doi:10.1371/journal.pntd.0009163)
Supplement: S1 Text — (DOCX) [file pntd.0009163.s003.docx]

**Supporting information S3: Annual tiny target deployments in the HAT active foci of Guinea**

**Boffa East**

First deployment: 2012

Number of tiny targets in 2018: 4,407

Tsetse density reduction in 2018: 57%

**Boffa West**

First deployment: 2016

Number of tiny targets in 2018: 3,875

Tsetse density reduction in 2018: 79%

**Forecariah**

First deployment: 2018

Number of tiny targets in 2018: 5,144

Tsetse density reduction in 2018: 71%

**Dubreka**

First deployment: 2016

Number of tiny targets in 2018: 2,653

Tsetse density reduction in 2018: 81%
